# Functional Anatomy of the Masking Level Difference, an fMRI Study

**DOI:** 10.1371/journal.pone.0041263

**Published:** 2012-07-27

**Authors:** David S. Wack, Jennifer L. Cox, Claudiu V. Schirda, Christopher R. Magnano, Joan E. Sussman, Donald Henderson, Robert F. Burkard

**Affiliations:** 1 Buffalo Neuroimaging Analysis Center, Dept. of Neurology, University at Buffalo, State University of New York at Buffalo, Buffalo, New York, United States of America; 2 Department of Nuclear Medicine, University at Buffalo, State University of New York at Buffalo, Buffalo, New York, United States of America; 3 MR Research Center, Department of Radiology, University of Pittsburgh, Pittsburgh, Pennsylvania, United States of America; 4 Department of Communicative Disorders and Sciences, University at Buffalo, State University of New York at Buffalo, Buffalo, New York, United States of America; 5 Department of Rehabilitation Science, University at Buffalo, State University of New York at Buffalo, Buffalo, New York, United States of America; University of Maryland, United States of America

## Abstract

**Introduction:**

Masking level differences (MLDs) are differences in the hearing threshold for the detection of a signal presented in a noise background, where either the phase of the signal or noise is reversed between ears. We use N0/Nπ to denote noise presented in-phase/out-of-phase between ears and S0/Sπ to denote a 500 Hz sine wave signal as in/out-of-phase. Signal detection level for the noise/signal combinations N0Sπ and NπS0 is typically 10–20 dB better than for N0S0. All combinations have the same spectrum, level, and duration of both the signal and the noise.

**Methods:**

Ten participants (5 female), age: 22–43, with N0Sπ-N0S0 MLDs greater than 10 dB, were imaged using a sparse BOLD fMRI sequence, with a 9 second gap (1 second quiet preceding stimuli). Band-pass (400–600 Hz) noise and an enveloped signal (.25 second tone burst, 50% duty-cycle) were used to create the stimuli. Brain maps of statistically significant regions were formed from a second-level analysis using SPM5.

**Results:**

The contrast NπS0- N0Sπ had significant regions of activation in the right pulvinar, corpus callosum, and insula bilaterally. The left inferior frontal gyrus had significant activation for contrasts N0Sπ-N0S0 and NπS0-N0S0. The contrast N0S0-N0Sπ revealed a region in the right insula, and the contrast N0S0-NπS0 had a region of significance in the left insula.

**Conclusion:**

Our results extend the view that the thalamus acts as a gating mechanism to enable dichotic listening, and suggest that MLD processing is accomplished through thalamic communication with the insula, which communicate across the corpus callosum to either enhance or diminish the binaural signal (depending on the MLD condition). The audibility improvement of the signal with both MLD conditions is likely reflected by activation in the left inferior frontal gyrus, a late stage in the what/where model of auditory processing.

## Introduction

The brain takes advantage of phase differences of binaural auditory stimuli to improve listening ability. An example is that a signal presented within a noise background can have greater than a 10 dB lower (better) detection threshold if it is presented out-of-phase rather than in-phase between ears, when the noise is interaurally in phase. The difference between these signal detection thresholds is known as a masking level difference (MLD). The contrasted stimuli used to determine MLDs can be identical in terms of intensity level, spectrum, and duration, yet the audibility of the signal is very different. The full neural network specific to the processing of binaural MLD stimuli is not well understood, nor has it been extensively investigated using functional imaging. We therefore utilize functional magnetic resonance imaging (fMRI), to localize neural regions involved in MLD processing.

Licklider (1948) [1], by altering the phase of speech presented in a noise background, found intelligibility highest with noise in-phase between ears, and the speech 180° (π radians) out-of-phase between ears. Hirsh (1948) [2] showed a detection advantage for a tone presented binaurally in background noise if the tone source was 180° out-of-phase between the two ears, compared to when both channels of the tone were in-phase. Following Hirsh’s work, psychoacoustic studies were performed to characterize the influence of the frequency of the signal, the bandwidth of the noise, and the phase and level differences between ears in affecting the magnitude of the MLD [2]–[14]. In an experiment that increased the masker from 5 to 65 dB SPL (sound pressure level), the MLD increased from 3.5 to roughly 15 dB [15]. The magnitude of the MLD has been found to decrease as the center frequency of the masker (signal frequency) increased [14], and increase with a decrease in the bandwidth of the masker. There is an increase in the magnitude of the MLD with an increase in the duration of the signal up to approximately 500 ms [3], [4], [16]. Interaural phase and time delay differences of the noise and signal have also been investigated. The best detection (lowest signal threshold level) occurred when either the signal or noise was 180° out-of-phase [5], [17].

We denote stimuli presented in-phase between ears with a 0, and π (radians) if presented out-of-phase. Both N0Sπ (noise in-phase, signal out-of-phase) and NπS0 (noise out-of-phase, signal in-phase) have a signal detection advantage compared to N0S0 (noise in-phase, signal in-phase). The MLD is the difference in the participant’s signal intensity threshold between MLD conditions, N0Sπ or NπS0, and the control condition N0S0. The MLD found with the N0Sπ-N0S0 comparison is typically larger (on the order of 2 dB) than NπS0-N0S0 comparison. Both are typically over 10 dB, and in individual cases can be greater than 20 dB [18], [19].

It is reasonable to expect auditory regions such as the inferior colliculus (IC) and auditory cortex (AC) to play an important role in the neural processing of MLD stimuli, and these regions have been investigated in animals. A series of studies by Jiang et al. [20], [21] and Palmer et al. [22] showed differences in neuron firing rates in the IC of the guinea pig related to S0 and Sπ, whereas the condition Nπ created little if any response, likely due to the de-synchronization of the stimuli. A study by Guo and Burkard [23] showed an increased near-field response in the auditory cortex (AC) of the chinchilla for MLD conditions compared to the control condition.

Human studies using electroencephalogram (EEG) recordings have indicated a cortical rather than brainstem MLD response. For example, Fowler and Mikami [24] showed that the slow vertex component P2 thresholds for signal detection in both N0Sπ and N0S0 conditions increased linearly with increasing noise level. N0Sπ increased with a smaller (better detection) slope than N0S0, consistent with the findings that the MLD increases for higher overall intensity levels. In a follow up study [25], Fowler and Mikami were unable to show an MLD in the middle latency response (MLR); the MLR is thought to arise from midbrain, thalamic and cortical regions of the auditory nervous system [26]. Wong and Stapells [27] used an amplitude-modulated signal component to evoke an auditory steady state response (ASSR), and found an MLD for the 7 and 13 Hz modulations rates for N0Sπ-N0S0, but not for NπS0-N0S0. Neither showed a difference at 80 Hz rates. Ishida and Stapells [28] were unable to find an MLD for the 40-Hz ASSR. These ASSR findings parallel the previous studies, if one accepts the view that ASSR modulation frequencies (>70–80) Hz result from superimposed brainstem response, those near 40 Hz reflect superimposed midbrain, thalamic and early cortical responses, and those of very low frequencies (<20 Hz) represent superimposed cortical responses.

Animal-based studies have investigated specific sites such as the IC and AC, whereas human-based psychoacoustic or evoked potential studies are less localizing. Dichotic listening studies, which use language-based tokens and require a participant to attend to a target in either ear, suggest a high level involvement (that includes the thalamus) may be required for processing the MLD. These studies often find a right ear advantage (REA) in attending to stimuli in the right ear, when competing tokens are presented in the left ear, as opposed to vice versa. Kimura proposed a model explaining REA, which included a right to left hemisphere crossing of auditory (speech) information [29]. Perceptually, differences in MLD conditions can be quite large, although acoustically the different MLD conditions are very close to each other, and typically only differ in the phase between ears of the noise or signal. Using these stimuli in an imaging study would allow the isolation and focus on the small differences between conditions.

Positron Emission Tomography (PET) and functional Magnetic Resonance Imaging (fMRI) imaging have been used to locate neural regions associated with auditory tasks, by comparing sequential image intensity values in relationship with changes in an auditory condition. In the case of H_2_0^15^ PET the changes in image intensity values are related to regional cerebral blood flow, in the case of BOLD fMRI the changes are due to changes in the oxygenation of the hemoglobin. Herein, we use the term “activation” to refer to a neural region which has statistically significant differences in image values for one condition versus another. Dichotic listening using consonant-vowel and musical-instrument stimuli together with the effect of attention was investigated by Hugdahl et al. [30]–[31] using ^15^O PET, which measured changes in oxygen utilization. This was followed by fMRI work which typically used dichotic word or syllable stimuli [32]–[38]. Budd et al. [39] used dichotic noise stimuli with varying levels of interaural correlation. Chait et al. [40] used MEG to study Huggins pitch (a dichotic pitch paradigm) and iterated ripple noise (a diotic pitch paradigm). Hall and Plack [41], [42] and Barker et al. [43] used fMRI to study these same stimuli and found activations in the auditory cortex. Puschmann et al. [44] used tones in noise (NπS0), Huggins pitch, binaural band pitch and Nπ noise, and also found pitch related activations in the auditory cortex. Ernst et al. [45] found regions that were mainly sensitive to the signal to noise ratio within and adjacent to lateral Heschl’s gryrus. A follow up study found regions in the auditory cortex related to co-modulation masking release [46].

In the present exploratory investigation, we postulate that listeners will have a regionally different neural activation in response to the MLD-dichotic conditions (e.g., N0Sπ) than to the MLD-reference (e.g., N0S0) conditions. Specifically, we will compare a listener’s BOLD level response to each of the MLD dichotic conditions: NπS0, N0Sπ, N0SL (noise in-phase, signal left ear only), and N0SR (noise in-phase, signal right ear only), to the BOLD level response while listening to the MLD reference condition N0S0 (i.e. NπS0 vs. N0S0, N0Sπ vs. N0S0, etc.). Because the present study is intended to be exploratory, we hypothesize, for the purposes of our analysis, that any brain voxel could show activation differences between MLD conditions. However, based on related MLD animal studies, we expect differences to be in more rostral regions of the central auditory nervous system, including the IC and AC. Furthermore, we conjecture there will be cortical differences in a listener’s BOLD response between the MLD dichotic conditions NπS0 and N0Sπ based on the commonly observed behavioral and EEG differences observed [27] between these MLD conditions.

## Methods

### Participants

The study protocol was approved by the University at Buffalo, Health Science IRB; all participants gave their informed written consent prior to auditory screening. Participants had to meet the following criteria: be between the ages of 18 and 45 years; be right-handed; have pure tone hearing thresholds of 25 dB HL or better for frequencies 250 Hz - 8000 Hz for each ear; and have a N0Sπ - N0S0 MLD of 10 dB or greater. Participants underwent screening and MLD threshold testing in a sound booth within a week prior to MRI testing.

### Auditory Testing

Signal threshold testing was performed in a sound booth for conditions: N0S0, N0Sπ, NπS0, N0SL, and N0SR. Threshold determination used a forced-choice design with three one-second length noise segments which were separated by.5 seconds and presented at 75 dB SPL. Participants had to determine which segment also included an enveloped 500 Hz tone as the signal. Testing started with a signal level of 85 dB SPL. This signal level stayed the same until the participant was able to correctly identify the signal two times in a row, or was unable to correctly identify the signal once. If the participant was unable to detect the signal, the signal level increased; if the participant was able to detect the signal in two successive trials at the same signal level, the signal level decreased. Seven direction changes were used. Step sizes between level changes were 8, 4, 4, 4, 2, 2, 2 dB. The average of the last two reversals was used as the threshold. MLDs were calculated by subtracting N0S0 threshold from the thresholds found for: N0Sπ, NπS0, N0SL, and N0SR. Participants additionally underwent a forced choice signal lateralization test, and identified randomly presented 1 second segments of N0SL, N0S0, or N0SR, as "Signal Left", " Signal Both", or "Signal Right" for 30 presentations. The tone signal was presented 3 dB above the participant's N0S0 signal threshold.

### Stimuli Construction

Conditions were created by summing together noise and signal segments using MATLAB (Natick, MA). The signal was a 500 Hz sine signal, presented in bursts lasting 250 ms with a 25 ms rise and fall time, presented every 500 ms. The noise for the presentations was created by sampling a very long duration of noise (approx. 10 minutes) created and filtered using a 400–600 Hz band-pass, equi-ripple finite impulse response (FIR) filter, with order 1064, having 50 dB attenuation +− 100 Hz, designed with the Filter Design and Analysis Tool in Matlab. The program used calibration values of noise and signal intensity as a program parameter. Calibration measurements were made using a Larson Davis System 824, with acoustic coupler AEC101 IEC 318 (LD-SLM) of individual noise and signal segments. The signal and noise were summed together by randomly choosing a point in the band-passed noise segment, and searching forward for 2 ms (one cycle of the signal) to determine the starting point that would give the highest correlation between the noise and signal. After selecting a starting point, one second each of noise and signal were summed together for the sound booth stimuli, and eight-second segments were summed together for the scanner stimuli. The noise used a.1 second ramp filter at the start and end of each stimulus.

### Acoustic Calibration

MRI-compatible headphones from Resonance Technologies, Inc. (Northridge, CA) were tested and levels calibrated using the LD-SLM. Signal phase between ears was checked using a single cycle sine wave as input to the headphones and was measured through the LD-SLM with an oscilloscope (Tektronix TDS3012). Based on our findings, the stimuli were corrected in software to compensate for reversal of phase (180°) by the headphones. The scanner headphone acoustic output was evaluated with a Stanford Research System Model SR785, Dynamic Signal Analyzer (DSA), to determine the frequency response to the noise and signal. In response to the 400–600 Hz band-pass noise, resonances in the acoustic system showed a maximum peak in the 600–700 Hz range. For this reason, a shortened version of the MLD testing was also performed with the scanner headphones to ensure that subjects had an MLD response with scanner headphones. The pure tone signal response of the headphones was also measured with the DSA, and didn't reveal any problems. Prior to each scanning session, presentation levels were verified using a Radio Shack model 33–2055 sound level meter, which was mounted to a fabricated coupler. Sennheiser 280 Pro headphones were used for the screening, and underwent similar testing and calibration. Correct phase of signal was observed between ears, as was a steep drop off below 400 Hz and above 600 Hz for the noise.

### Scanner Room MLD Testing

MLD threshold testing in the scanner room using scanner headphones followed the same procedure used in the screening (described above), but used only five direction changes, and only two conditions: N0S0 and N0Sπ. This testing was used only for assessment of MLD effect size in the scanner environment. Signal level for fMRI presentation was determined separately using longer length segments such that participants could barely identify that the signal was present for the N0S0 condition, but could not identify if the signal was present 2 dB lower. This signal level was fixed for all stimulus conditions.

### Scanner Conditions

Scanner conditions were "NoStim" (No Stimuli), N0, Nπ, S0, Sπ, N0S0, N0Sπ, NπS0, N0SL, and N0SR. Each presentation lasted for eight seconds. Presentations followed one second of quiet, which was inserted to prevent an adaptation effect between scanner coil noise and stimuli. The scanner TA (acquisition time) was 3 seconds, resulting in 12 seconds between the start of consecutive conditions. Four sessions were collected for each subject. Each session presented each of the 10 conditions six times. The conditions were presented in randomly-chosen permutations with the provision that neighboring permutation end and start conditions could not be the same. Prior to each session, participants were instructed to "listen for the signal". After each session, participants were asked whether they were still comfortable. Each session included three frames prior to the stimuli presentation, which were discarded.

### Scanning Parameters

MR Imaging was performed using a GE 3T Signa Excite HD 12.0 Twin Speed 8-channel scanner (General Electric, GE, Milwaukee, WI) with a maximum slew rate of 150 mT/m/ms and maximum gradient amplitude in each orthogonal plane of 50 mT/m (zoom mode). An 8-channel head coil (In Vivo Corporation, Orlando, FL) was used for all acquisitions. A high-resolution 3D fast spoiled gradient echo (FSPGR) scan was collected at a voxel size of 1×1×1 mm (acquisition matrix of 256×256, FOV 25.6 cm). 174 locations per slab were acquired, 1 mm thick, ensuring whole brain coverage. Echo/repetition time for the 3D FSPGR scan were TE/TR = 4.1/9.1 ms, flip angle = 20, 1 average, and bandwidth 19.23 kHz (150 Hz/px). 2D sparse functional imaging was performed with a TE = 35 ms. and group delay  = 9 seconds, which provided nine seconds of “quiet”, during which the stimuli was presented, followed by TA = 3 seconds during which the fMRI planes were actively acquired [47], [48]. 27 slices 4 mm thick were acquired with no gap, using a 128×128 acquisition matrix, and FOV = 24.0 cm, for an in-plane resolution of 1.9 mm×1.9 mm.

### Processing

Dicom image files were converted to NIfTI format using dcm2nii (MRIcron; http://www.sph.sc.edu/comd/rorden/mricron/). Realignment and co-registration of functional images to the participants T1 weighted image was performed using SPM5 (http://www.fil.ion.ucl.ac.uk/spm). Segmentation was performed on the participant's T1 image, which provided spatial normalization parameters for transforming the co-registered functional images into the coordinate system of the provided SPM templates. Scans were smoothed using an 8×8×8 mm Gaussian kernel. After using SPM5 to create the general linear model including all conditions for each session, SPMd [49] was used to identify outlying scans. Next, a first -level SPM analysis was then performed for each subject which included all four sessions, but excluded scans identified by SPMd as having greater than 30 times the median number of outlier voxels, or which had more than 1 mm total motion from the previous scan. The first-level analysis provides separate images showing activation for each individual for each contrast. A second-level analysis was then performed using the contrast images produced for each individual in order to make statistical parametric maps.

## Results

Five female and five male participants were recruited. All met the inclusion criteria described above for the study. Male and female participants matched in age within 2 years and ranged in age from 23 to 43 years; the mean female and males ages were 29.2 and 29.8 years, respectively. After each scan session, all participants were alert when spoken with, and reported being comfortable. Each participant completed the full scanning session.

### Auditory Testing Results

Mean threshold values for N0S0, N0Sπ, NπS0, N0SL, and N0SR measured during the auditory testing performed in the sound booth were 68.6, 54.2, 56.4 59.0, and 59.0 dB SPL, respectively. Hence, the mean MLD for N0Sπ –N0S0 and NπS0– N0S0 were 14.4 and 12.2 dB, respectively. Mean MLDs for N0SL and N0SR were both 9.6 dB, but participants had up to an 8 dB imbalance between these two conditions. The N0SR-N0S0 MLD for participant 4 was zero. The mean N0Sπ - N0S0 MLD measured using scanner headphones was 11.4 dB, with minimum and maximum values of 4 and 16 dB. Individual MLD thresholds are given in Table 1, a comparison of the MLD thresholds measured in the soundbooth and scanner room is given in Table 2.

**Table 1:** Thresholds (measured in dB SPL) for detection of 500 Hz sinusoid signal in 75 dB SPL, 400–600 Hz band-pass noise, with all measurements made in the sound booth.

Participant	Gender	Age	N0S0 (Ref)	N0Sπ (MLD)	NπS0 (MLD)	N0SL (MLD)	N0SR (MLD)
1	M	31	67	55 (12)	55 (12)	57 (10)	57 (10)
2	M	27	63	49 (14)	55 (8)	59 (4)	59 (4)
3	F	43	67	55 (12)	55 (12)	57 (10)	61 (6)
4	F	26	65	55 (10)	55 (10)	59 (6)	65 (0)
5	F	30	73	61 (12)	59 (14)	59 (14)	65 (8)
6	M	25	67	53 (14)	55 (12)	59 (8)	57 (10)
7	F	24	77	53 (24)	57 (20)	59 (18)	57 (20)
8	M	25	71	57 (14)	61 (10)	61 (10)	57 (14)
9	M	41	65	51 (14)	53 (12)	59 (6)	51 (14)
10	F	23	71	53 (18)	59 (12)	61 (10)	61 (10)
Mean		27	68.6	54.2 (14.4)	56.4 (12.2)	59.0 (9.6)	59.0 (9.6)

**Table 2:** A comparison between MLD condition measurements (in dB SPL) made in the sound booth and scanner room of the thresholds for detection of 500 Hz sinusoid signal in 75 dB SPL, 400–600 Hz band-pass noise.

Subject	N0S0	N0Sπ	ScannerMLD	Sound Booth MLD
1	63	55	8	12
2	67	53	14	14
3	63	55	8	12
4	61	57	4	10
5	73	57	16	12
6	65	53	12	14
7	67	51	16	24
8	71	59	12	14
9	65	53	12	14
10	67	55	12	18
	66.2	54.8	11.4	14.4

For the lateralization testing, four participants had a 90% success rate or better, and three participants had a 30% success rate or worse (i.e. less than expected by chance). However, in less than 4% of responses did participants mistake the signal presented to the left as right, or vice versa. Since the signal was presented at 3 dB above the N0S0 threshold, it was on average ∼13 dB above the thresholds for N0SL and N0SR. Individual lateralization results are provided in Table 3.

**Table 3:** Individual volunteer’s confusion matrices for identifying whether the signal was presented to the “Left”, “Both”, or “Right” ear(s).

Participant No.	Lateralization % Correct	Response	Presentation		
			Left	Both	Right
1	90.0%	Left	4	2	0
		Both	0	10	1
		Right	0	0	13
2	23.3%	Left	0	2	2
		Both	3	6	12
		Right	1	3	1
3	93.3%	Left	6	0	0
		Both	0	12	1
		Right	0	1	10
4	26.7%	Left	4	1	3
		Both	1	3	5
		Right	0	12	1
5	40.0%	Left	3	5	2
		Both	1	3	5
		Right	1	4	6
6	63.3%	Left	1	0	1
		Both	3	10	2
		Right	0	5	8
7	76.7%	Left	4	2	0
		Both	1	9	1
		Right	0	4	9
8	30.0%	Left	6	10	1
		Both	4	1	6
		Right	0	0	2
9	93.3%	Left	6	0	0
		Both	0	12	0
		Right	0	2	10
10	90.0%	Left	5	1	0
		Both	0	12	0
		Right	0	2	10

### MRI Results

In Table 4 we describe all second-level analyses that meet the strict criterion of significance p<.05 correcting for family-wise error (FWE), and regions that meet a weaker "trending" criterion of p<.1, FWE, which for comparison was roughly equivalent to p<.00001 uncorrected, for voxel-wise comparisons.

**Table 4:** Table of SPM activations for study contrasts.

Contrast	MNI Coor.	Location Label	Size	Cluster P, FWE	Voxel P, FWE
	x,y,z mm		mm^3^		
N0Sπ-N0S0	−52, 30, 10	LIFG	170	0.737	0.017
N0S0-N0Sπ	33,−7, 20	R. Insula	1608	<0.001	0.757
NπS0-N0Sπ	−34, −7, 21	L. Insula	2919	<0.001	0.809
	31, −22, 21	R. Insula	433	0.091	1.000
	10, 69, 14	SuperiorFrontal Gyrus	513	0.047	0.676
	14, 27, 10	Genu CorpusCallosum	566	0.031	0.999
	10, 13, 16	Corpus Callosum	1476	<0.001	0.386
	26, −24, −2	R. Pulvinar Thalami	2241	<0.001	0.201
N0Sπ – N0SL	−8, −82, −15	Declive	101	0.955	0.051
N0SR-N0Sπ	−22, −3, 23	Left Caudate Nucleus	3618	<0.001	0.754
	27, 26, 18	Right caudate Nucleus	5835	<0.001	0.826
	18, −14, 37	Right Cingulate Gyrus	536	0.027	0.351
N0-N0Sπ	50, 11, 14	Post. Central Gyrus	551	0.041	1.000
	63, −53, 13	R. STG	465	0.081	0.836
NπS0-Nπ	−47, 31, −3	LIFG	932	0.056	0.988
	24, −32, 9	R. Pulvinar Thalami	575	0.025	0.999
N0-Nπ	−42, 60, 44	Left Angular Gyrus	627	0.023	0.919
	51, −59, 34	Right Angular Gyrus	861	0.004	0.982
	4, −34, 31	Right Cingular Gyrus	1012	0.002	0.817
	10, −57, 26	Right Posterior Cingulate	456	0.084	0.813
	7, 43, 25	Right Medial Frontal Gyrus	1239	<0.001	0.982
	54, 24, 8	Right Inferior Frontal Gyrus	852	0.005	0.955

For each region of activation; the values from SPM are reported, together with the best representative anatomical label.

### MLD: N0Sπ vs. N0S0

Second-level random effects analysis revealed a small region reaching voxel-wise significance in the left inferior frontal gyrus (LIFG) for the contrast N0Sπ - N0S0. The opposite contrast N0S0 - N0Sπ showed a 1608 mm^3^ region of significant activation, located in and around the right insula.

### MLD: NπS0 vs. N0S0

Second-level analysis did not reveal any regions reaching significance using FWE correction for the contrast NπS0– N0S0. The maximum occurred within the LIFG, p<.0001, uncorrected, which we include in our discussion because of its similar location to the N0Sπ –N0S0 activation. Likewise the opposite contrast, N0S0-NπS0, did not reveal any significant or trending regions. The largest threshold region was located in left insula and planum polare (p = .167, FWE, cluster-wise).

### N0Sπ vs. NπS0

**Figure 1:**
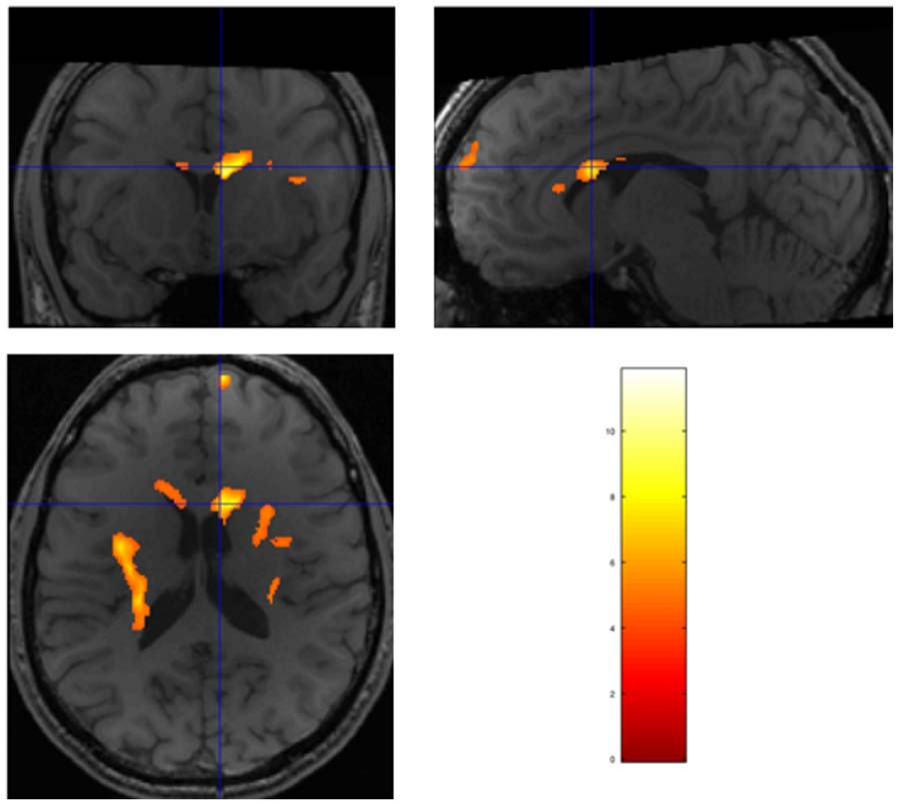
Two separate activation regions are seen within the corpus callosum for the group comparison using the 2^nd^ level contrast NπS0 - N0Sπ, threshold p<.001, uncorrected for family-wise error (FWE). Because the activation maps have been smoothed at 8×8×8 mm FWHM, there may appear to be an overlap of the activation with the ventricle seen on the high resolution T1 image, which we did not attempt to mask. The statistics for this cluster is an indication that there exists at least one point of activation within the cluster with true significance (p<.01, FWE, cluster-wise for the larger region, Table 4). Obviously, the true source of activation would be in the tissue region, as the region of significance would likely be near voxels with the highest t-scores, which are located within the corpus callosum. The large activation seen in the L. insula is also cluster-wise significant (p<.01). All conditions were presented using random permutation ordering to prevent possible cyclic responses or habituation effects being mix with the contrast of conditions. Furthermore, SPMd was used to prevent any possible influence of outliers or motion.

**Figure 2:**
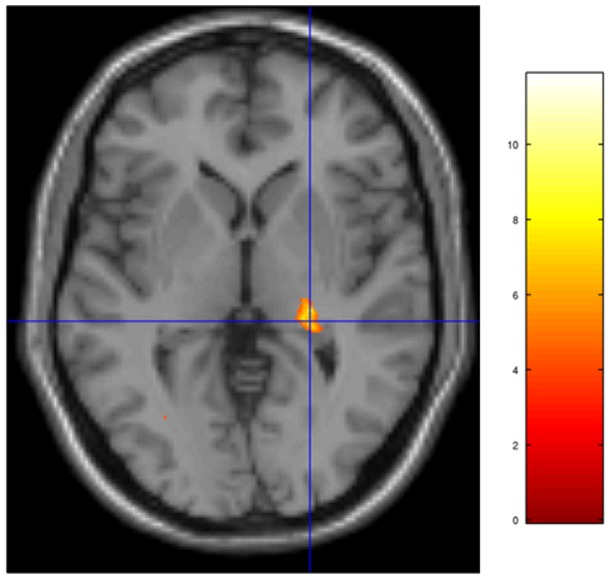
Activation seen in the right pulvinar thalamus for the group comparison 2^nd^ level contrast NπS0 - N0Sπ, threshold p<.001, uncorrected for FWE.

The contrast NπS0 - N0Sπ had a widely-distributed set of regions which reached significance: left insula, right superior frontal gyrus, a region on the right side of the corpus callosum, and the right pulvinar thalami; three of these regions had significance p<.01, FWE, cluster-wise. Additionally, the right insula met our weaker cluster significance threshold of p<.1, FWE. Statistical parametric maps showing the (group) activation in the corpus callosum and pulvinar thalamus are shown in Figures 1 and 2. There were no regions which reached or approached significance for the contrast N0Sπ - NπS0.

### Lateralization

SPM results for conditions involving N0SR and N0SL did not exhibit clear patterns. The contrasts N0SL vs. N0S0, N0SR vs. N0S0, and N0SL vs. N0SR had no regions of activation. The most notable results arose from the contrast N0SR – N0Sπ, which showed large activations in and around the left and right caudate nucleus. Individual participant results for contrasts involving N0SL and N0SR vs. N0S0 appeared inconsistent. We believe this might be due to differences in subjects' lateralization ability. To test this belief, an analysis using a participant's overall lateralization success (percentage correct) as a covariate was attempted, as was limiting the analysis to only participants who performed well on the lateralization task. The results of both post-hoc analyses did not reach significance, and likely suffered from limited power.

### Other

We also present comparisons between three noise-with-signal conditions versus the corresponding noise-only conditions: N0S0 vs. N0, N0Sπ vs. N0, and NπS0 vs. Nπ. We found no activations between N0S0 and N0, in either direction. The opposite contrast, N0-N0Sπ, yielded two regions: one in the post-central gyrus that reached significance, and the other in the right STG that approached significance. The contrast NπS0-Nπ revealed two regions that approached or reached significance: LIFG (similar in location to the contrasts NπS0-N0S0 and N0Sπ –N0S0), and right pulvinar thalamus. There were no activations for the opposite contrast, Nπ -NπS0.

**Figure 3:**
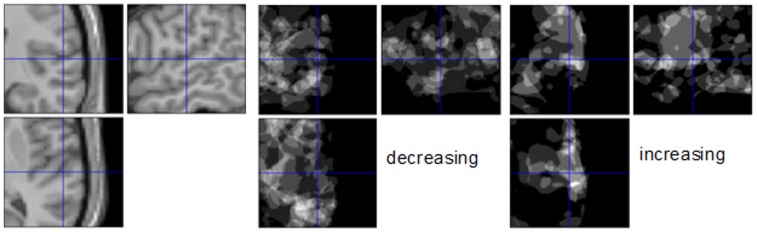
Left image: T1 image showing R STG. Middle image: subjects with a decrease in activation for N0Sπ – N0S0. Right image: Image of number of participants with an increase in activation for N0Sπ – N0S0.

We hypothesized, but did not observe, activation in the primary auditory cortex with the N0Sπ – N0S0 contrast. In Figure 3, we show the number of participants that presented increased activation in the R. STG (SPM T >1.0), as well as the number of participants showing decreased activation (SPM T <1.0). The cross hairs mark a location in the right STG where three participants exhibited an increase, three a decrease, and four had no change for the contrast N0Sπ – N0S0. For the N0Sπ-NoStim contrast, a search of voxels with p<.01 (uncorrected) reveals a cluster of 3012 mm^3^ in the left STG, which is cluster-wise significant, p = .035, FWE. There was a cluster located in the right STG, which did not reach significance with peak spm t value  = 5.5 and extent size of 1620 mm^3^. For the contrast N0S0– NoStim there was a cluster with extent size 1207 mm^3^ and peak spm t = 5.19 in the left STG, and a cluster with extent size 1187 mm^3^ with peak spm t = 5.44 in the right STG, however neither cluster was statistically significant accounting for FWE. As a check of the processing, the contrasts S0– NoStim was examined for all subjects using a height threshold of p = .01, uncorrected. All but two subjects had a peak spm t value >3 located in both the left and right STG, with peak spm t = 11.5. While no regions were significant for the second level analysis, there was a cluster of size 1100 mm^3^ with peak spm t = 5.8 located in the left STG, and a cluster with extent size 2185 mm^3^ and peak spm t = 8.12 located in the right STG, using a height threshold of p<.01, uncorrected. Hence for our tested Stim vs NoStim contrasts, we consistently observed clusters of voxels with moderately high spm t values located in the STG, as would be expected in response to auditory stimuli.

## Discussion

Our study used functional imaging to search nearly the entire brain for neural correlates to the MLD. We did not find support for our hypothesis of activation associated with MLD (comparisons: NπS0 vs. N0S0, N0Sπ vs. N0S0, N0SL vs. N0S0 and N0SR vs. N0SR) in more rostral regions of the central auditory nervous system, such as the IC and AC, which was based on animal model work [20]–[23]. We do not rule out the involvement of these regions, but speculate that the functional anatomical variability of the AC prevented detection using voxel-wise statistics family-wise error corrected across the whole brain. However, our results do indicate clear neural correlates of the MLD in the insula, pulvinar thalamus, and corpus callosum. We interpret this activation pattern as consistent with the Kimura model for REA for speech processing [29], and syllable-based dichotic-listening studies [36]–[38], [50]–[54].

The main assumptions of Kimura's model are: 1) auditory information is principally processed in the temporal lobe contralateral to the ear of presentation; 2) the left hemisphere is more specialized for language/speech processing than the right (in particular for right-handed participants); 3) there is a decussation of auditory information from the right hemisphere across the corpus callosum to the left hemisphere (which is specialized for the processing of speech stimuli) for further processing; and 4) the ipsilateral pathway can be suppressed by the contralateral pathway [29], [55].

Assumption 1 is firmly established in the literature [56], but is not addressed by our data, as all of our conditions (except the NoStim condition) are presented to both ears. Assumption 2 is supported by our data, as there was a significant cluster for the contrast N0Sπ -NoStim with extent size 3012 mm^3^ which reached significance within the left STG, whereas a cluster about half the size located in the right STG did not reach significance. The activation in the LIFG for the MLD contrasts: N0Sπ - N0S0, NπS0– N0S0, and NπS0-Nπ also fits with the left lateralization proposed by the “what” portion of the “what”/“where” model [57]–[60], which postulates that the neural processing of information will follow different pathways, depending on whether it is being processed based on recognition or localization. While the Kimura model is for speech, and we used tonal (500 Hz) stimuli, it is not unreasonable to expect a left-hemisphere dominant response, since the stimuli were not continuous but presented in short 250 ms enveloped bursts every half second. The left AC has been shown to respond well to temporal changes [55], as would be required in the tracking of formants. We did not find significant regional activation in the right STG, for any of the contrasts using the “NoStim" condition or any other evidence to argue for right hemisphere dominance.

Activation of the corpus callosum for the contrast (NπS0– N0Sπ) gives evidence of inter-hemispheric communication (part of assumption 3 of the Kimura model). While less common, corpus callosum activation has been previously observed, including in studies that involved stimulation requiring high inter-hemispheric communication [61]–[63]. We were careful to guard against artifacts, and we believe this white matter activation to reflect a true processing path. The two contrasted conditions are similar perceptually compared to the other stimuli, and we did not observe any relative motion of the subjects between conditions. While we employed standard SPM realignment methods, we also eliminated scans that had more than 1 mm total motion from the previous scan. The conditions were presented in an order determined by random permutations, and hence all conditions were balanced in being presented both early and late in the presentation sequence. The contrasts in this study were all “within” subject, hence we do not expect an artifact due to spatial normalization differences, such as could be found if comparing between groups. Finally, we used SPMd to eliminate scans that had the possibility of being a transient, which was a cautionary step most others do not take, likely because of the increased difficulty of performing the analysis.

We note that differences between our MLD conditions NπS0 and N0Sπ imply an underlying activation difference between at least one of the conditions and the control condition, N0S0. The noise portion of the stimuli has a wider bandwidth and a (generally) higher overall SPL than the sine-wave portion. Accordingly, we speculate that metabolic differences in processing are largely influenced by changes in the noise component of our stimuli. We conjecture that information of the noise signal for NπS0 crosses the corpus callosum, going from right to left hemisphere. We believe this ipsilateral (double crossing) noise signal may combine with the matching contralateral signal in the left insula, with a net suppression effect. Plausible evidence supporting this belief is seen by the activity decrease in the left insula for NπS0 compared to N0S0.

**Figure 4:**
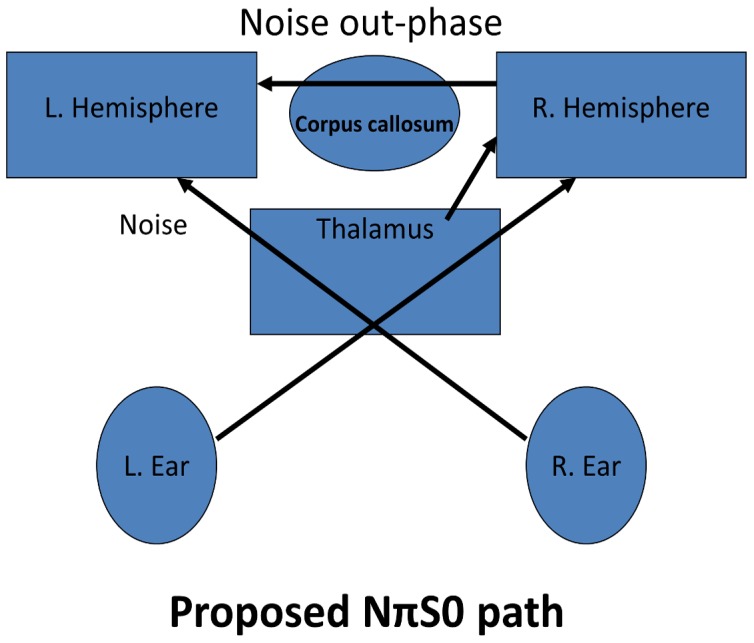
Diagram showing theorized release from masking processing paths for NπS0. When the noise portion of the stimuli from the left ear arrives in the left hemisphere after crossing the corpus callosum, the subsequent combined noise component after mixing with matching but opposite-phase noise from the right ear is suppressed, because the combining signals lack coincidence.

**Figure 5:**
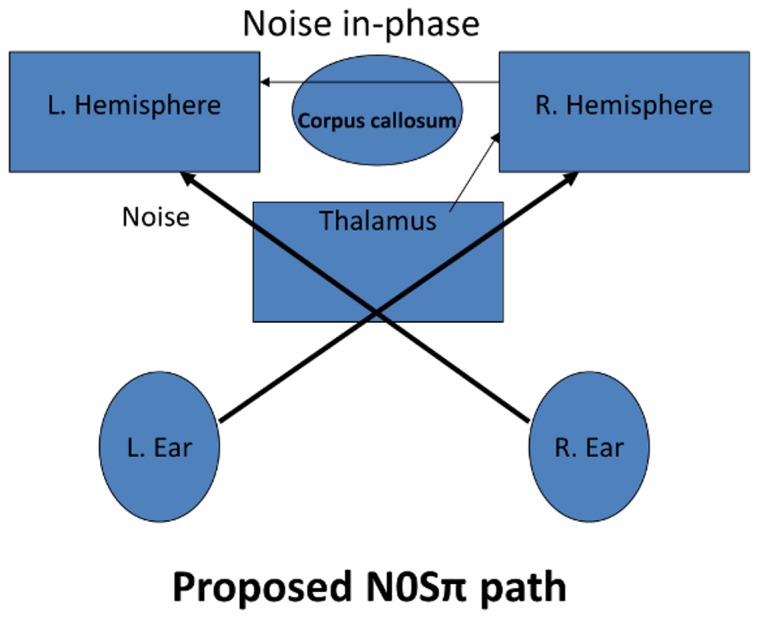
Diagram showing theorized release from masking processing paths for N0Sπ. When (if) the noise signal from the left ear arrives in the left hemisphere, after crossing the corpus callosum, it has already been greatly suppressed. The site of the original suppression could be at the right pulvinar thalamus or insula of the right hemisphere.

In contrast, we conjecture that the noise portion of N0Sπ is suppressed earlier in the processing chain, perhaps in the right insula or pulvinar thalamus, which would support the reduced activity seen in the corpus callosum (contrast: NπS0– N0Sπ), and the large decrease in activation (1500 mm^3^) in the right insula (contrast: N0S0 - N0Sπ). Furthermore, we believe that the resulting combination of the noise signal with the ipsilateral auditory signal in the left insula is reduced as a result of the diminished inter-hemispheric transfer. Diagrams of our hypothesized release from masking models for the MLD conditions NπS0 and N0Sπ are shown in Figure 4 and 5. While dichotic listening is generally believed to involve the transfer of auditory information across the corpus callosum, studies which have examined the effects of surgical sectioning of the corpus callosum indicate that primary auditory pathways are more towards the caudal end than the activation we found [64]–[66] This could indicate that the contrast reflects a decrease in activity for the N0S0 condition. This is consistent with previous findings, where one subject [64] had an improved score for a left ear attention after the anterior sectioning of the corpus callosum. If nothing else, the contrast difference between NπS0– N0Sπ, shows that all dichotic stimuli are not treated similarly. The surgical studies used dichotic speech pairs (numbers or constant vowels). We suspect that auditory signals cross in the caudal portion of the corpus callosum, but the *differences* between conditions were not great enough to be observed. Again, white matter activation is rare, and the reason we found a difference may only be because we are observing both a slight increase and decrease compared to the control condition N0S0.

Assumption 4 of Kimura’s model states that the ipsilateral pathway is suppressed; which has empirical support from the study by Pollmann et al. [50], who found that patients with lesions in the posterior part of the corpus callosum showed a nearly 100% REA. Our data is not inconsistent with this, but neither does it support this element of the model.

### Dichotic Listening

The thalamus has been proposed as a gating system for speech (and possibly other stimuli) to be sent to more rostral brain regions [67], [68], based on dichotic-listening experiments with patients undergoing stereothalamotomy. A dichotic-listening study by Fitch et al. [69] found that lesions in the posterior thalamus inhibited the processing of auditory stimuli, including attending to stimuli presented to a particular ear. If the pulvinar thalamus is acting as a gating mechanism in our study, we propose that it is triggered when the signal is present in the stimuli. The right pulvinar was observed as part of the large activation pattern for the contrast NπS0 - N0Sπ (Table 4 and Figure 1). The results also revealed activation in the pulvinar thalamus for the contrast NπS0– Nπ (p = .025, FWE, cluster-wise), which gives another example of its responsiveness to the presence of the signal in background noise. As a final example, if the signal is removed from the contrast of MLD conditions NπS0-N0Sπ (which had strong activations), the resulting contrast, Nπ-N0, has no regions with significant activation. The reverse contrast, N0-Nπ, yielded a very different activation pattern, with significant activation found in the left and right angular gyrus, right cingular gyrus, right posterior cingulate, right medial gyrus, and right inferior frontal gyrus.

### Lateralization

Our study's focus was on finding neural correlates associated with the MLDs, and we have reported on the primary focus of the study. In addition to the MLD conditions N0Sπ and NπS0, we used the MLD conditions N0SL and N0SR. These conditions have an interesting place in the hierarchy of the MLD conditions, since starting from the N0SL or N0SR condition, the addition of the signal in-phase to the opposite ear becomes N0S0, or becomes N0Sπ if the added signal is π radians out-of-phase. Based on pilot testing, we believed that participants would be able to distinguish between the signal being presented to the left, right or both ears, amid the noise background.

Lateralization testing with MLDs is not normally performed, and has only infrequently been reported in the literature [70]. The results of the lateralization testing, which was only performed during the screening session (in the soundbooth) were very mixed, with four participants performing very well, and three very poorly (slightly less than expected by chance). Yet, overall, in less than 4% of responses did participants mistake the signal presented to the left as right, or vice versa. The signal was presented at 3 dB above the N0S0 threshold, and was on average ∼13 dB above the thresholds for N0SL and N0SR. The thresholds for N0SL and N0SR were also mixed. For example, one subject’s MLD for N0SR was 0 dB. By basing the signal level on the N0S0 condition, we effectively made the lateralization harder on those who may have had a more effective strategy for N0S0 signal detection. The remainder of MLD behavioral testing was unremarkable. For example, we observed N0Sπ was roughly 2 dB better than NπS0 [14], which was roughly 2.5 dB better than N0SL or N0SR. Based on the lateralization results, we will assume that at least some of the participants were unable to lateralize the location of the signal while in the scanner. The perceptual difference for a signal presented in-phase and out-of-phase diminishes at levels above threshold [71], therefore we did not consider a higher signal level.

Lack of a strong finding in our hypothesized regions of AC and IC has some support from previous studies. We believe our results to be consistent with a study by Hall and Plack [41], [42], which used dichotic stimuli to investigate Huggins pitch, where the perception of pitch was created by linearly changing the phase between ears through 1 cycle of a small band centered around 200 Hz of broadband noise. In their study, Huggins pitch was contrasted against a “just-noise” condition, whereas we contrasted our MLD conditions against N0S0, which has a detectable pitch due to the presence of the (audible) in-phase 500 Hz stimulus. As both MLD and non-MLD conditions had a detectable tonal stimulus in our study, we expect our ‘pitch’ vs. control contrast to be smaller than that found by Hall and Plack. In a study using 16 participants, Hall and Plack had comparisons between pitch and noise that did not identify a single pitch center common to all listeners [41] (pg. 579). However, as an indication of the between-subject anatomical variability of the auditory cortex, they were able to find regions sensitive to pitch stimuli in most subjects, but in slightly varying locations. Hence, our lack of finding any MLD-related activation in the AC is not surprising. In our study we had fewer subjects, and we limited ourselves to FWE statistics corrected for the whole brain as a search region. The advantage of our approach is that we were able to find regions we didn’t originally specify (e.g. pulvinar thalamus); our disadvantage is that our analysis methods are less sensitive than studies that limit their search to the auditory cortex.

The MLD conditions, when compared to the no stimulus condition (N0S0 - NoStim and N0Sπ - NoStim), only showed one cluster which was significant, which was located in the left STG for contrast N0Sπ – NoStim. However, for both contrasts, there were clusters in both the left and right STG with peak spm t value greater than 5 and size greater 1100 mm^3^, when using a height threshold of p = .01, uncorrected. Examination of individual results for contrast S0– NoStim showed that there was large variability in responses between individuals ranging from two subjects having only a weak activation in either the left or right STG to one subject that had peak t values greater than 11 (bilaterally). We believe that the weakness of these contrasts is likely the result of anatomical variability. Also, given that our instructions to the subject was to “listen for the signal”, different subjects may have treated the absence of stimuli ambiguously [72]. We’ll note that we consider our true control condition for the study to be N0S0, and that the “NoStim” condition was intended primarily to test the processing path. We believe that our finding for N0S0– NoStim in which the signal portion (S0) is barely audible, is similar to that by Hart et al. [73], who on a larger data set (12 participants versus our study’s 10 participants, 28 repetitions vs. our study’s 24, and using a presentation level of 90 dB SPL compared to our presentation level of 75 dB SPL), reported no activation for stationary unmodulated (i.e. constant) tones.

Our study compared conditions that were the same in intensity, spectrum, and duration, and we expected neural activation differences because of the perceptual differences. Yet the conditions used in the contrasts with the two largest activation patterns, NπS0 - N0Sπ and N0 - Nπ, are close enough that perceptually they may be hard for some to distinguish, in the same way that some may not recognize stereo speakers or headphones as being wired out of phase. Our rationale for comparing NπS0 and N0Sπ was based on the findings of Wong and Stapells [27], who found an auditory steady state response MLD for modulation frequencies of 7 or 13 Hz for the N0Sπ versus N0S0 comparison, but not for the NπS0 vs. N0S0 contrast. That we find greater activation for NπS0 than N0Sπ, yet the auditory evoked response MLD was seen only for N0Sπ, could be due to the auditory evoked response being sensitive to the signal portion of the stimulus (i.e., phase locked to the envelope of the signal), while the fMRI finding was driven by the noise portion of the stimulus, as we previously argued.

### Strengths and Weaknesses

Our design approach was purposely broad (using 10 conditions) and exploratory in nature. Utilizing fMRI, we were able to search nearly the entire brain for activation patterns in response to MLD conditions. The benefit of our broad approach was that we achieved strong and interesting results outside of our stated study hypothesis, while within our SPM analysis hypotheses. We opted against the use of a button press for monitoring a participant’s attention in order to avoid potential conflicting neural activations; participants were instead instructed to listen for the signal. We believe this approach was sufficient for our set of attentive and well-intentioned participants. The participants, as observed through conversation with the scanners communication system, remained alert throughout the study. SPMd was used as a final guard to identify and eliminate scans which may have been influenced by system transients or brief, unexpected participant behavior or focus.

A sparse MRI sequence allowed the stimuli to be presented during periods of relative quiet, and provided a better environment for listening for the modulated signal in the noise. We included a one second gap of ‘no stimulus’ between the end of the scanner data collection and the presentation to preserve a clear and consistent onset of the stimulus, and to prevent an auditory adaptation effect [74], [75] from the scanner noise. Since the band-passed noise component of the stimuli and 500 Hz tone are correlated, we went through the additional step of searching through 2 ms of the noise, to find the noise starting position that gave the maximum correlation, for a consistent presentation strategy [76]. The N0S0 threshold with the scanner headphones in the scanner room was approximately 2 dB better (lower) than in the sound booth. The lower threshold is likely related to a peak in the noise spectrum above 600 Hz for the scanner headphone, which implies a lower overall noise level near 500 Hz for the scanner vs. sound booth headphones. Also, this indicates that the use of a sound booth isn't critical, likely due to the 75 dB SPL background noise masking much of the environmental background noise.

Our study enrolled 10 participants, and was limited by resources. This allowed sufficient power for our primary hypothesizes and contrasts, as many of our reported regions had p<.01, FWE, cluster-wise. Our analysis of a hypothesized region in auditory cortex showed that some participants had increased activations while some others had decreased activations. We believe this indicates that a moderate increase in the number of participants would not have appreciably improved our findings for our hypotheses. However, while we approached the analysis globally, a regionally-specific analysis of the auditory cortex (which also accounted for anatomical variability of the auditory cortex) may have been able to find significance. While we are satisfied with not using a button press for our main hypothesizes, we recommend that future work, if it focuses on lateralization, include a button press.

### Conclusions

Our findings reveal a network of neural correlates associated with the MLD (that are outside of the previous focus of MLD research) which involves the pulvinar thalamus, the insulae, and a neural process that crosses the corpus callosum. These findings, in particular the involvement of the pulvinar thalamus, fit with the dichotic listening research, and are congruent with the proposed model of Kimura.
